# Primary care physicians’ views on osteoporosis management: a qualitative study

**DOI:** 10.1007/s11657-019-0599-9

**Published:** 2019-04-26

**Authors:** Helena Salminen, P. Piispanen, E. Toth-Pal

**Affiliations:** 1 Division of Family Medicine and Primary Care, Department of Neurobiology, Care Sciences and Society, Karolinska Institutet, Stockholm, Sweden; 2 Academic Primary Healthcare Centre, Region Stockholm, Stockholm, Sweden

**Keywords:** Focus group interviews, Qualitative study, Osteoporosis, Fragility fractures, Primary care physicians

## Abstract

**Summary:**

Osteoporosis is an under-diagnosed condition; only around 14% of patients in Sweden receive bone-specific treatment after a fragility fracture. This qualitative interview study found that primary care physicians perceive osteoporosis as a silent disease that is overshadowed by other conditions and is complicated to manage.

**Purpose:**

To explore primary care physicians’ views on managing osteoporosis.

**Methods:**

A total of 17 primary care physicians in Stockholm participated in four focus group interviews. Interview transcripts were analysed with thematic analysis.

**Results:**

One main theme was found: *Osteoporosis—a silent disease overshadowed by other conditions*. The main theme contained five sub-themes. Physicians perceived osteoporosis as a low-priority issue. They described uncertainty about managing it and insufficient awareness of the condition in primary healthcare (PHC). Physicians had differing opinions about who is responsible for managing osteoporosis. They reported that the health care system regulated their work such that they gave low priority to the condition. They were uncertain about the value of the Fracture Risk Assessment Tool (FRAX). The physicians thought that financial incentives, education, and increased collaboration with other relevant health care professionals and with patients were needed to increase the priority of osteoporosis in PHC.

**Conclusion:**

Physicians perceived osteoporosis as a silent disease that is complicated to manage. They gave low priority to osteoporosis and thought their patients shared this view. The physicians saw other issues and medical conditions as more important than osteoporosis. They wanted better collaboration at their PHC centres and with hospitals. They also wanted district nurses to be more involved in managing osteoporosis and especially in assessing fracture risk.

## Introduction

Lack of appropriate testing and treatment for patients with osteoporosis and fragility fractures is a worldwide problem [1–5]. “Osteoporosis care of fracture patients has been characterised as the Bermuda Triangle made up of orthopaedists, primary care physicians and osteoporosis experts into which the fracture patient disappears” [6]. Fragility fractures result in pain, suffering, and disability and are costly to society [7–9]. Sweden has one of the highest incidences of such fractures in the world, but only about 14% of patients with a diagnosed fragility fracture are treated with bone-specific drugs in the 12 months after their fracture [7]. According to the National Guidelines from 2012, primary health care (PHC) has the main responsibility for patients with osteoporosis and for following up fragility fractures in Sweden [10]. Deficiencies in transferring information about fractures from hospitals to PHC contribute to under-treatment with bone-specific drugs, and thus, to insufficient secondary prevention. One way to close this gap might be to establish Fracture Liaison Services (FLS) [5, 11, 12].

The Fracture Risk Assessment Tool (FRAX) was introduced to identify people at high risk of fragility fractures. The tool (https://www.sheffield.ac.uk/FRAX/index.aspx) [13–16] integrates several risk factors to calculate 10-year absolute fracture risk. In Sweden and elsewhere, FRAX is the recommended tool for determining whether further investigation into osteoporosis is warranted. Nevertheless, we know little about primary care physician’s thoughts about the tool and its use.

Healthcare professionals’ attitudes towards osteoporosis management might help explain poor management of the condition [17–20]. However, there are many more qualitative studies about patients and their attitudes towards osteoporosis than about health professionals’ attitudes [2, 21–25]. In a study that compared patients and PHC physicians’ perspectives about adherence to osteoporosis medication, the researchers found that physicians were worried about structural barriers to care, costs, adherence to medication, side effects, and the reliability of information patients obtained on their own [26]. The physicians thought patients lacked knowledge about osteoporosis, whereas the patients thought both they and their PHC physicians lacked sufficient knowledge [26]. Few qualitative studies have investigated how healthcare professionals’ attitudes influence osteoporosis management in primary care [17–19], but a group of Australian researchers found that primary care physicians ranked osteoporosis as less important than other conditions, such as diabetes, osteoarthritis, cardiovascular disease, and hypertension [19]. They sometimes thought the guidelines were not clear (e.g. about treatment duration) and worried that the cost of medication would be problematic for patients. However, they were confident that the medications were effective. In a previous study, we also found that district nurses in Sweden often felt frustrated with the management they could provide for patients with osteoporosis [17].

The aim of this study was to explore primary care physicians’ views on osteoporosis management in PHC.

## Subjects and methods

### Design

The design was a qualitative study with focus group interviews.

### Context

In the Swedish PHC system, all PHC centres have contracts with county councils and receive their funding on the basis of these contracts. There are around 200 PHC centres in Stockholm County, and 70% of them are privately run. Information about fragility fractures diagnosed at hospitals is not automatically passed on to PHC centres, and patients with fragility fractures are rarely referred to PHC for osteoporosis assessment. At the time of this study (2013 to 2014), the Stockholm County Council had not yet fully implemented the Swedish National Board of Health and Welfare’s 2012 guidelines for osteoporosis care. There was no systematic FLS; only a few FLS projects had been carried out. The same reimbursement regulations apply for bone-specific drugs as for any other medication in Sweden, which means that there is a cap on patients’ annual out-of-pocket prescription costs.

### Participants

Participants were recruited both by advertisement and by e-mail invitation to specialists and medical residents in family medicine at all PHC centres in Stockholm County. A total of 17 physicians from 14 different PHC centres volunteered to participate. Four focus group interviews were conducted. Three had four to six participants. One had two participants because of last-minute cancellations. In one of the focus groups, all participants were residents.

Before the focus group interviews, the participants filled in a questionnaire on their backgrounds. Eleven of the 17 participants were women. Three had worked as specialists in family medicine for more than 10 years and four for fewer than 10 years. Ten of the participants were residents.

One of the questions asked how many times the physician had diagnosed a patient with osteoporosis during the last 12 months. Three responded that they had done so 11 to 20 times, 13 that they had done so less than 11 times, and one, that they had not done so at all. When asked how often they used FRAX, six answered that they used it at least once a month, the others less often or never. Eight of the participants reported that they had sufficient knowledge about osteoporosis, but 15 of the 17 said that they would like to learn more about it.

### The focus group interviews

The focus group interviews lasted for 60 to 90 min. A semi-structured interview guide was used. It was designed to facilitate the discussion by stimulating thoughts and ideas on the topic. The same interview guide was used in all the interviews, but one minor adjustment was made after the first interview. The interview guide covered the following areas: potential to improve care, barriers to improving care, and responsibility for the care of patients with osteoporosis and fragility fractures. There were also some questions about FRAX and the participants’ opinions about the tool.

Each interview was led by a moderator, and an observer was present. The observer’s role was to support the moderator and to observe the interactions among the participants. The observer took field notes, which were reviewed after each session. The observers were specialists in family medicine but were not clinically active when the study was conducted. The moderator was a physiotherapist, which increased the triangulation in the research process. All of the interviews were audio recorded and transcribed verbatim.

### Data analysis

The focus group interviews were analysed with thematic analysis [27]. After each interview, the moderator and observer discussed what had emerged during the focus group interview, reading the notes taken by the observer. To become familiar with the data, each member of the research team read the transcripts in their entirety. Each researcher then performed an initial coding by condensing the text and identifying and selecting important data elements. Next, they organised codes into meaningful groups to form sub-themes and themes. Codes, sub-themes, and themes were then discussed by the whole group, re-analysed, and refined until consensus was reached about the meaning and structure of the sub-themes and themes that best reflected the meaning of the data.

### Ethics

The Ethical Review Board in Stockholm approved the study (2013-1782-31-4). All participants received written and oral information about the study prior to giving written informed consent to participate.

## Results

**Fig. 1:**
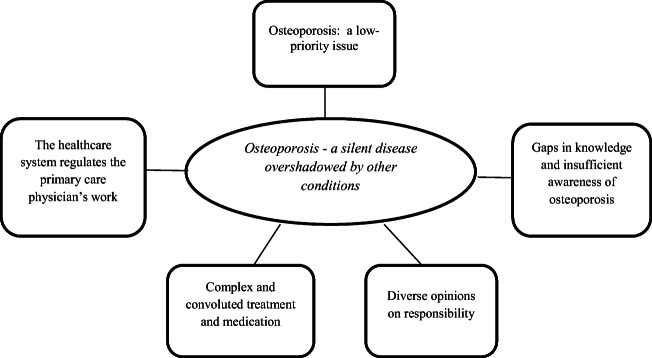
The main theme and the five sub-themes from the analysis of the group interviews

One main theme emerged from the analysis: *Osteoporosis—a silent disease overshadowed by other conditions*. The main theme described both the manifest and latent content found in the data, and it consisted of five sub-themes. Figure 1 presents the structure of the main theme and the sub-themes.

The physicians described osteoporosis as a distant matter that hardly ever crossed their minds. Participant Y said, “It’s not likely that my brain will think, ‘Let’s start an inquiry into whether you have osteoporosis or not’; it’s so distant in a way” (4.297–298). Other medical conditions, like diabetes or heart disease, had higher priority. Their experience was that patients also prioritised other medical conditions because they rarely brought up osteoporosis. L: “And it is also, as several of you already mentioned, that they [patients] seldom come and visit us to discuss osteoporosis, that they want an assessment for it. And then it happens easily that you don’t think of it either” (3.53–54). The physicians worked under time constraints that made it difficult for them to address any but the most prioritised issues. As a result, they seldom took the initiative to handle issues other than those brought up by the patient, even though they would have preferred to provide more holistic patient care. Additionally, management of osteoporosis was neither prioritised on the organisational level nor in the reimbursement system. F: “I mean, if we get a referral and we need to make a decision about it, then it will get done. . . . If they [the hospital] don’t send it, then we don’t get the information.” G: “But is not Sweden . . . among the countries that have the highest frequency of osteoporosis-related fractures? Why hasn’t anything happened earlier then if there’s such under-diagnosis and under-treatment? I find it strange. What’s casting a shadow over it?” K: “It’s a systemic error, I think” (3.963–977).

1. *Osteoporosis: a low-priority issue*

The primary care physicians mentioned many reasons for why osteoporosis was not a priority for them. Other diseases were more important because they had immediate, severe consequences, e.g. heart conditions, diabetes, and cancer. D: “I think that patients with osteoporosis that often becomes manifest in old age . . . those patients often have significant comorbidity that contributes to making the problem [osteoporosis] invisible.” E: “Yes, most often everybody is more anxious about a myocardial infarction, stroke, and cancer.” B: “Osteoporosis doesn’t cause many symptoms . . . not in the same way as angina pectoris or whatever else” (2.203–219).

The patients also prioritised other conditions. N: “I think that one of the difficulties is that the patients don’t visit the doctor with the question whether they have osteoporosis or not. And it’s not the first question I think of as a doctor, because these patients do have other issues that are more important.” M: “For them [the patients] it’s not actually a disease to have had a fracture . . . so you have to actively enquire about whether they have had one” (1202–275). The patients with osteoporosis were mostly older women, who are often silent and accommodating in the healthcare system. E: “It’s mostly elderly women, the ones who have less say and lowest priority . . . why not engage an assistant nurse to watch . . . that a referral has been sent [to PHC] before the patient is discharged [from the hospital]” (2.332–333)? Some of the physicians also regarded them as too old for bone-specific treatment. The physicians also felt uncertain about how to deal with both older patients and younger immigrants with low vitamin D levels. Some of the participants described another problem that was very common some years earlier when younger postmenopausal women who were afraid of developing osteoporosis insisted on extensive unjustified investigations. Often they had become alarmed after having read articles about osteoporosis in the mass media. M: “I think there was a lot about osteoporosis in the newspapers then, . . . then there were many healthy, very well-kept women who came and had concerns, that took time”(1. 223–240). There were, though, two physicians, both specialists, who expressed awareness of and interest in osteoporosis and who did not perceive it as problematic to handle.

2. *Gaps in knowledge and insufficient awareness of osteoporosis*

The physicians discussed gaps in knowledge about osteoporosis and the management of fragility fractures at all levels in the healthcare system, among patients, and in society in general. The participants reasoned that more informed and educated patients would increase demand for osteoporosis assessments. R: “Make sure there’s information in the waiting room. Leaflets you can take home. So the question of osteoporosis will be raised.” T: “And write an article in the medical newspapers about osteoporosis management.” Y: “And, in addition . . . actively educate patients” (4.880–888). “S: I think there’s a lack of knowledge and information among healthcare professionals in general, nurses and doctors, but also among different levels of managers who create the rules about priority [in health care]” (3.884–887). L: “I do think that both patients and the doctors should be more aware, because then they will remind us and even demand more from us” (3.1526–1527). The physicians perceived that patients with immigrant backgrounds knew even less about osteoporosis than patients with Swedish backgrounds.

There was consensus among the participants that knowledge about and awareness of osteoporosis and the management of fragility fractures needed to increase, both in the population in general and among patients and healthcare professionals. L: “I need to increase my own knowledge . . . how many fractures can you prevent? . . . How effective are those drugs and what kinds of side effects do they have? What is the cost-benefit for the patients?” F: “I have heard, but I don’t remember, 60 percent was it for some . . . and higher for another . . . risk reduction” (3.860–871).

Although only a few of the participants had not heard of FRAX, and most of them knew how it worked, several of them were unsure how to interpret the results. The more experienced physicians chose not to use FRAX because it took valuable time from the patient encounter, and they perceived it as an extra burden of uncertain value. D: “[I] very seldom [use FRAX] . . . it doesn’t contribute much to the work we’re doing . . . FRAX isn’t crucial for the medication the patient is about to get.” E: “We can evaluate FRAX – it’s not that fantastic – it has many drawbacks. But it’s good enough to move the assessment further along” (2.671–685).

In all the interviews, participants said that if FRAX is to be used regularly, it ought to be linked to the electronic medical records. Y: “It is a very good instrument. It would be great if all those instruments were integrated in the medical records” (4.237–238). They also suggested that district nurses at the PHC centre could be responsible for FRAX. P: “Yes, I actually also thought about it right now, that FRAX could be something the patients fill out before the visit, or together with the nurse. Like we do with screening for chronic obstructive pulmonary disease” (4.486–487).

In general, residents had a more positive attitude towards FRAX than the more experienced specialists in family medicine.

3. *Diverse opinions on responsibility*

Participants had diverging opinions about where the main responsibility for osteoporosis management lay. Many knew that according to the guidelines, PHC physicians were supposed to bear the main responsibility, with support from other specialists such as orthopaedists and geriatricians. R: “It belongs to primary care, definitely . . . but if there’s a manifest fracture, then I think that you can also require the hospital to help us.” Y: “I think it’s everybody’s responsibility. . . . Then maybe we have a big part of this responsibility, but it’s everybody’s responsibility.” S: “But it can belong more specifically to us when we initiate some treatments . . . for example, treatment with cortisone.” Y: “Yes . . . then it’s our responsibility. But most often, they’ve been in the hospital first. And then I think they should also think about it” (4.323–342). Most of the participants said that the clinic where the fracture was diagnosed, most often in a hospital, should be responsible for starting assessment and treatment of the patient. This could include measuring bone density with DXA, initiating bone-specific medication, or sending a referral to PHC. K: “Primary care, of course [has the main responsibility].” L: “But if there’s a fracture, then it is the orthopaedist.” F: “Exactly. The one who notices the fracture, whoever it is.” L: “But I think also that anyone who initiates treatment with cortisone, which raises the risks for the patient in some way, they have to take responsibility, too.” G: “In my opinion, it should be a must to always send a referral” (3.1236–1252).

Several physicians said that the main responsibility for getting adequate assessment and treatment lay with the patients themselves. E: “The primary care physician [has the main responsibility].” D: “Yes, the primary care physician.” C: “I think that the patient has the main responsibility for themselves.” E: “Yes, of course. After the patient” (2.493–499).

At the PHC centre level, the participants wanted to share the responsibility for these patients with the district nurses. N: “I believe in a district nurse with special interest and knowledge about osteoporosis, like these district nurses with knowledge about diabetes and asthma and chronic obstructive pulmonary disease . . . so that we can get a template filled in with all of the patient’s risk factors . . . that they do the groundwork for us.” M: “I believe, as you said, in the district nurse as ‘the spider in the web’” (1.482–493). They mentioned that studies had shown that collaboration with nurses improved the quality of care for many patient groups in primary care, such as patients with diabetes and hypertension. They thought that patients with osteoporosis would also benefit from such teamwork. Additionally, they thought that the district nurses could assist them by coordinating the management of these patients and by assessing the patients with FRAX before the doctor’s appointment. G: “The district nurses maybe could have a questionnaire . . . the patient could start with meeting a nurse . . . then we can make the decision whether to do FRAX, for example.” F: “Yes, I think they could do FRAX, too, and then we can make the decision about whether to send them for a DXA” (3.179–186).

The primary care physicians also wanted to increase collaboration with physicians in hospitals, such as geriatricians and orthopaedists. The participants thought a structure, such as the FLS, would make it easier to collaborate with other specialist physicians and with district nurses.

4. *Complex and convoluted treatment and medication*

The physicians were concerned about the side effects of bone-specific drugs and about patients’ adherence to treatment. They were worried about the oesophageal problems that bisphosphonates can cause and assumed that their patients did not like the medication. The patients told them that weekly administration of bisphosphonates interfered with their daily routines. Only a minority of the physicians had heard of annual infusions with zoledronic acid, and hardly any had prescribed them. C: “And then you aren’t even able to get rid of the substance [infusions] from your body, and I’ve totally stopped with that kind of treatment, when you consider what you might cause . . . when we actually don’t know” (2.476–480). They doubted the effectiveness of bone-specific drugs, especially in older patients, and thought the harmful side effects outweighed the benefits. Many participants described it as time consuming and complicated to explain to patients how they should take the bone-specific drugs and said following up the treatment was also difficult. Some of them doubted the effectiveness of the medication on the basis of their observations of often minimal increases in bone mineral density. They were uncertain about how long they should treat a patient with bone-specific drugs and how to assess the need for calcium and vitamin D supplementation. D: “If you’re talking about bisphosphonates that have been on a patient’s medication list for longer than eight years, then you should . . . terminate it.” E: “And now they don’t know any more, maybe five years; but three years has been also mentioned. So . . . we don’t really know” (2.853–861). The physicians thought the existing guidelines did not provide sufficiently clear guidance. Most had heard about serious side effects of bisphosphonates, such as osteonecrosis of the jaw and atypical fractures, and were afraid to put their often frail patients at risk of these kinds of serious complications.

5. *The healthcare system regulates the primary care physician’s work*

The participants described how their daily work was ruled by financial incentives in the healthcare system and how they did what their clinic was paid for them to do. C: “The financial matter is an obstacle” (2.106). Some measures were promoted and received extra reimbursement, but managing osteoporosis was not one of them. R: “The only thing that could work would be a quality bonus, then you can mark that it is done, and then it would be desirable to find those patients.” T: “Yes, let’s say that if you do a FRAX with a patient, than you get a quality bonus. . . . Then people [healthcare professionals] would begin to do it.” S: “Yes, very likely.” Y: “That would change a lot” (4.425–435).

According to participants, some PHC centre managers also instructed physicians to limit the number of issues discussed during each visit. S: “We’re directed to talk about only one issue during each visit, and that is what they [the patients] made the appointment for” (4.393).

The participants reported that DXA assessments and laboratory tests were financially burdensome for their centres. Including osteoporosis in the quality assurance and bonus system would relieve this burden and increase the priority given to the condition. They mentioned diabetes as an example of bonuses in the quality assurance system leading to high-quality care.

## Discussion

This qualitative study investigated and described primary care physicians’ views about managing patients with osteoporosis. The main finding was that they perceived osteoporosis as a silent disease. The primary care physicians rarely thought about osteoporosis and perceived it as complicated to manage.

Our interpretation of the results of this qualitative study is that many factors contribute to the low priority of osteoporosis in PHC. The healthcare system has not resolved the question of who is responsible for initiating the assessment of a patient after a fragility fracture or of how to build a functioning FLS organisation in which PHC the role of PHC is clear. At the time of the study, implementation of national guidelines for osteoporosis care in PHC had failed in many respects. Hospitals rarely assessed patients after a fragility fracture and seldom referred them to PHC.

Our results on the low priority of osteoporosis are in line with those of previous studies of healthcare professionals and patients [2, 17, 19, 21–26, 28]. In an Australian study, general practitioners prioritised other conditions but had greater trust in the efficacy and safety of the treatment than the participants in our study [19]. The complexity of osteoporosis management became obvious during the interviews in our study, as the participants expressed uncertainty about diagnosing and treating osteoporosis and perceived the guidelines as unclear. Furthermore, their patients rarely came to visit their PHC doctor to discuss osteoporosis.

Researchers in the UK who interviewed women about their views on having a diagnosis of osteoporosis found that the women ranked their other diseases as more severe [22]. Those findings are in agreement with our findings that primary care physicians thought patients prioritised other conditions. The patients who would benefit the most from treatment—older adults who had experienced fragility fractures—often had many other medical conditions that both physicians and patients prioritised over osteoporosis.

Participants reported that the cost of assessing osteoporosis with DXA scans and laboratory tests was higher than the reimbursement from the county council, which meant a net financial loss for the PHC centre. They thought FRAX took valuable time from the consultation with the patient and was not readily available because it was not integrated in the electronic medical record system. These factors, which kept osteoporosis off the agenda during the consultation, were the result of shortcomings in the healthcare system at the macro level, in the PHC organisation at the meso level, and in the actions of the individual physicians and patients at the micro level. The results of our study shed light on primary care physicians’ concerns about caring for patients with osteoporosis and on barriers to providing such care. To help ensure that PHC professionals take the primary responsibility for patients with osteoporosis and for following up fragility fractures, future guidelines and future FLS for caring for patients with osteoporosis need to take these macro-, meso-, and micro-level concerns into account. Our study indicates that factors at all levels contribute to maintaining the low priority of osteoporosis. At the macro level, there are national guidelines for osteoporosis, which recommend FLS, but no organised effort has been made to implement them in PHC. Our finding that the physicians had diverging opinions about who is responsible for taking care of patients with osteoporosis illustrates this. At the meso level, the reimbursement system for PHC in Stockholm includes bonus payments to centres for taking care of specific diagnoses (e.g. diabetes) but not osteoporosis. This has made the PHC centres focus their efforts on other diagnoses. At the micro level, individual physicians often described uncertainty about how to assess, treat, and follow up patients with osteoporosis. Suggestions and requests that participants brought up—a specialist nurse for handling patients with osteoporosis at the PHC centre and the systematic transfer of information from hospitals on patients with fragility fractures—are also elements in FLS. This highlights the importance of prompt implementation of the national guidelines.

Most of the physicians in our study were concerned about the safety and effectiveness of bone-specific drugs. These findings are in agreement with those of a study of 2515 general practitioners in the UK that showed that about two-thirds of the practitioners had doubts about the efficacy of bone-specific drugs [28]. Additionally, patients with osteoporosis often share these worries, as shown in previous studies in which patients have been interviewed [29].

Physicians in our study reported the same main reasons for not using FRAX as family physicians and general internists in a previous study [30], including poor access to clinical decision support systems. Decision support tools need to be easily accessible and user-friendly to achieve their intended level of use [31]. Because FRAX was not linked to the electronic medical records, participants in our study perceived it as a task that took extra time and were thus not prepared to use it. A Belgian study also found limited use of FRAX in PHC [32].

A previous study from researchers at the University of Karlstad described a Swedish model of structured interprofessional caretaking that resulted in an improved chain of care for patients with osteoporosis [33]. We also found that physicians wanted more interprofessional collaboration in managing osteoporosis in PHC. The participants in our study anticipated that care for patients with osteoporosis would improve if district nurses were generally more involved in managing the condition and were specifically responsible for performing fracture risk assessment.

Most of the primary care physicians in our study intended to provide holistic, patient-centred care, but because patients rarely brought up concerns about osteoporosis, this condition was seldom the focus of the encounter.

## Strengths and limitations

All participating physicians were clinically active at the time of the study, and they worked at 14 different PHC centres; both factors increase the credibility of the study. Both male and female physicians took part, and participants varied in age. All participants could be described as credible, and all had experience managing patients with osteoporosis in PHC. Although the sample size was small, we could see saturation in the analysis; i.e. no new issues were discussed in the last interviews. One potential limitation was that study participants were probably more interested in osteoporosis than their colleagues who chose not to participate.

We attempted to achieve trustworthiness throughout the whole process by following the steps of thematic analysis [27]. Initially, all researchers analysed the material independently, defining their own themes and sub-themes, and later we worked to achieve consensus by comparing the themes. The research group included different professions, which allowed us to triangulate our findings during the analytical process. The observers were specialists in family medicine, whereas the moderator was a physiotherapist who had no connection to primary care. In our opinion, this was an advantage because the moderator had no preconceptions about the primary care physicians’ statements. This created a relaxed atmosphere where participants could freely share their thoughts. The transferability of the results should be reasonably high because managing osteoporosis and fragility fractures is also a challenge for PHC in many other countries.

## Conclusions

This study showed that primary care physicians perceive osteoporosis as a silent disease and as complicated to manage. They gave low priority to osteoporosis and thought their patients shared this view. The physicians saw other issues and medical conditions as more important than and therefore overshadowed osteoporosis. They wanted better collaboration at their PHC centres and with hospitals. They also wanted district nurses to be more involved in managing osteoporosis and especially in assessing fracture risk.

## Abbreviations

FLS: fracture liaison service
PHC: primary healthcare
